# Re-evaluating a Prenatal Do Not Attempt Resuscitation Order in a Neonate With Ebstein’s Anomaly: A Case Report Highlighting Ethical Flexibility in Neonatal Care

**DOI:** 10.7759/cureus.94890

**Published:** 2025-10-18

**Authors:** Atheer Alhalawi

**Affiliations:** 1 Respiratory Therapy, King Faisal Specialist Hospital and Research Centre, Riyadh, SAU

**Keywords:** ebstein’s anomaly, ethical decision-making, neonatal ethics, neonatal resuscitation, prenatal dnar, resuscitation policy

## Abstract

Ebstein’s anomaly is a rare congenital heart defect with a variable prognosis. This report presents a male neonate with a prenatal do not attempt resuscitation (DNAR) order due to a presumed poor prognosis from complex congenital heart disease (CHD). Unexpected postnatal stability led to a re-evaluation of the diagnosis and the reversal of the DNAR status to full support. This case highlights the importance of dynamic assessment, multidisciplinary collaboration, and ethical consideration in neonatal care decisions.

## Introduction

Do not attempt resuscitation (DNAR) orders in neonates with complex congenital anomalies are typically based on expected survival and quality of life. Ebstein’s anomaly is a rare congenital heart disorder, occurring in approximately one out of every 200,000 live births and accounting for less than 1% of all congenital cardiac anomalies [1]. Normally, the tricuspid valve separates the right lower heart chamber (right ventricle) from the right upper heart chamber (right atrium (RA)). In patients with Ebstein’s anomaly, the positioning and function of the tricuspid valve are abnormal, resulting in impaired separation between the chambers [2]. However, postnatal clinical outcomes may deviate from prenatal assumptions, necessitating re-evaluation of care goals. This case describes a rare scenario in which a prenatal DNAR order was reversed following clinical and diagnostic reassessment in a neonate affected by this malformation.

Ebstein’s anomaly results from apical displacement of the septal and posterior tricuspid valve leaflets, which creates “atrialization” of a portion of the right ventricle and reduces effective right ventricular (RV) contractility. The condition is associated with significant tricuspid regurgitation, cardiomegaly, and a predisposition to arrhythmias, including supraventricular tachycardia and Wolff-Parkinson-White syndrome. Clinical severity varies widely: neonates may present with profound cyanosis and heart failure requiring urgent intervention, while others remain stable with mild symptoms and may survive into adulthood. Prognosis largely depends on the extent of valve displacement and associated anomalies, with some patients requiring surgical repair early in life, while others demonstrate prolonged survival with conservative management.

## Case presentation

A male infant was born at 37+5 weeks of gestation with antenatally diagnosed Ebstein’s anomaly and dysmorphic features (low-set ears, retrognathia, short neck, micropenis, hypospadias, and hydrocele). Antenatal echocardiography demonstrated a complex congenital heart disease (CHD) consistent with Ebstein’s anomaly, showing a dilated right atrium, severe tricuspid regurgitation, poor contractility, and evidence of pleural effusion and ascites. These findings indicated poor prognosis and led to a prenatal do not attempt resuscitation (DNAR) decision. A DNAR order had been signed before birth due to the anticipated poor prognosis.

**Figure 1:**
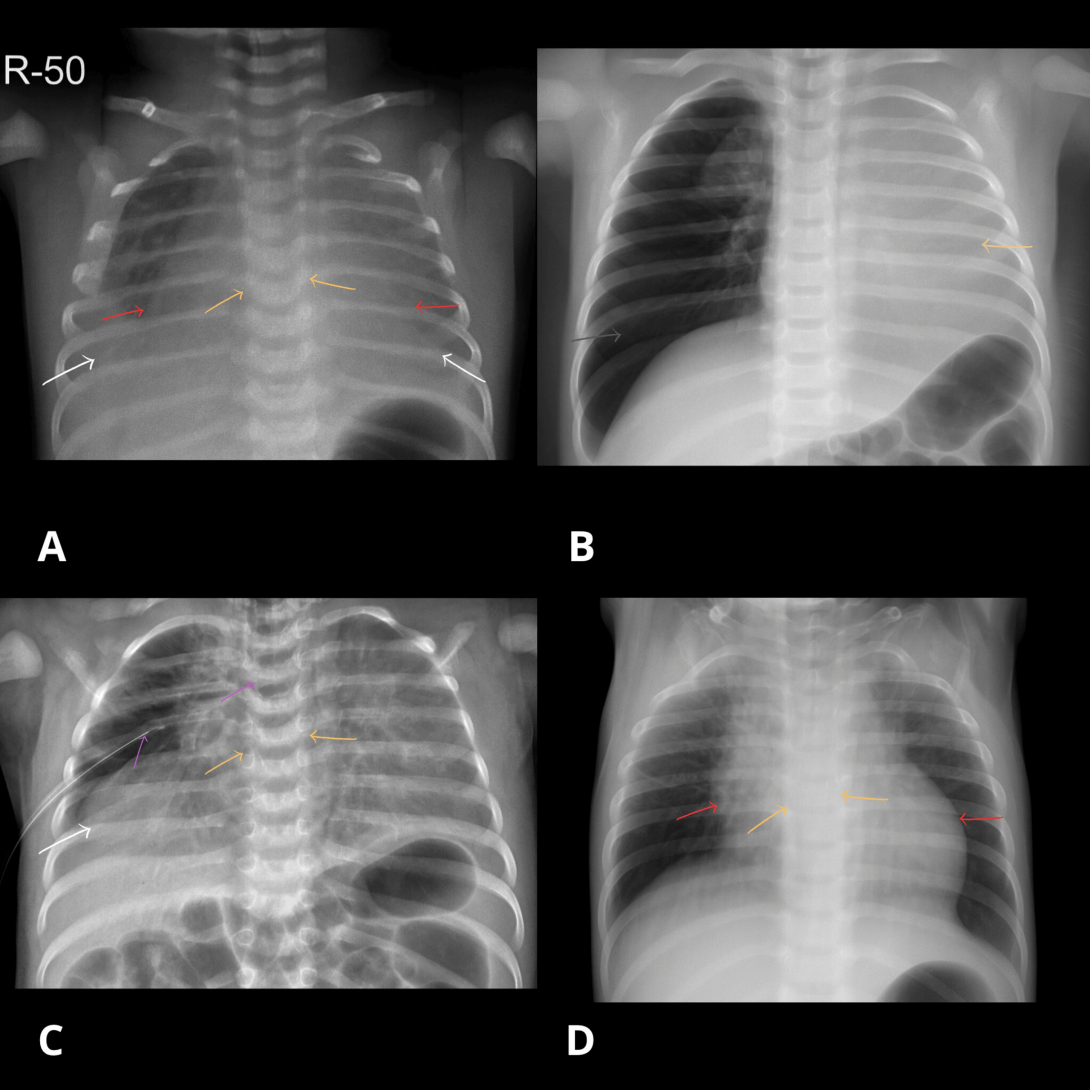
Serial chest radiographs in a neonate with Ebstein’s anomaly Chest radiographs before and after intervention, annotated with colored arrows. A: Pre-intervention chest X-ray. B: Post-intubation chest X-ray (endotracheal tube initially high, later repositioned). C: Post-intervention chest X-ray following chest tube insertion. D: Follow-up chest X-ray after clinical improvement and removal of the endotracheal tube and chest tube. White arrows: Blunted costophrenic angles, consistent with pleural effusion. Orange arrows: Silhouette sign, loss of the normal lung-cardiac border. Red arrows: Enlarged cardiac borders, suggesting cardiomegaly. Gray arrow: Right-sided pneumothorax. Yellow arrow: Opacified left lung with absent aeration. Pink arrow: Endotracheal tube. Purple arrow: Chest tube. Images were enhanced for clarity. No diagnostic elements were altered.

After delivery, the neonate maintained oxygen saturation at 88% on room air but showed grunting, prompting neonatal intensive care unit (NICU) admission. He was started on 2 L/minute nasal cannula oxygen. Chest X-ray revealed severe pleural effusion (Figure 1).

On day 2, a multidisciplinary meeting involving pediatric cardiology and cardiac surgery concluded that the baby had a mild form of Ebstein’s anomaly, with moderate to severe tricuspid regurgitation but adequate pulmonary artery forward flow. A repeat echocardiogram performed by the pediatric cardiology team confirmed a mild form of Ebstein’s anomaly with moderate to severe tricuspid regurgitation and good pulmonary artery forward flow, prompting the reversal of the DNAR status to full code. The patient was started on non-invasive ventilation (NIV).

On day 3, the patient developed worsening respiratory distress associated with pneumothorax and persistent pleural effusion. He was intubated, and a chest tube was inserted. Pneumothorax was most likely secondary to barotrauma in the context of underlying lung compromise. The patient remained intubated for eight days, as the persistent pleural effusion continued to impair lung expansion and gas exchange. He was then extubated to NIV and later weaned to continuous positive airway pressure (CPAP). Although transitioned to high-flow nasal cannula, a follow-up chest X-ray demonstrated worsening lung condition related to unresolved pleural effusion, which necessitated escalation back to CPAP. With the addition of chest physiotherapy to improve lung aeration and diuretic therapy as an adjunct to reduce further fluid accumulation, gradual clinical improvement was achieved. The patient was weaned to a low-flow nasal cannula on 1 L/minute at a fraction of inspired oxygen (FiO₂) of 21% (room air) via blender, maintained stability and was ultimately off supplemental oxygen.

## Discussion

This case underscores the complexity of decision-making in neonatal cardiopulmonary conditions and the importance of postnatal reassessment following prenatal DNAR decisions. Some infants with CHD do well at delivery, requiring no more than routine assistance such as drying, warming, and stimulating. In one report of 110 infants with prenatally diagnosed CHD, additional resuscitation was necessary in only 11 [3]. Nearly 75% of neonates born in stable condition with Ebstein’s anomaly can be managed with supplemental oxygen and prostaglandin infusion, with close observation for adequacy of cardiac output and oxygen saturation levels [4].

For neonates with Ebstein’s anomaly, cone repair has become the preferred valvuloplasty beyond the neonatal period, offering long-term durability in various anatomical presentations. When suitable, it should be considered early in childhood to prevent further cardiac dilation. Cone repair following RV exclusion may effectively balance early survival in this challenging lesion, avoiding the long-term risks of single-ventricle palliation [5].

Critically ill neonates with Ebstein’s anomaly can be successfully treated using RV/RA exclusion combined with a modified Blalock-Taussig (BT) shunt in cases where RV function is poor [6]. Severe Ebstein’s anomaly in neonates remains a challenging condition [7].

Do not attempt resuscitation (DNAR) decisions in neonatal intensive care units are made only after thorough discussions with parents or legal guardians. These discussions must include a clear explanation of prognosis and the potential outcomes of resuscitation and are subject to revision if the infant’s condition changes significantly [8].

Postnatal echocardiography is necessary to confirm and further define the anatomical and physiological features of congenital heart defects diagnosed in utero. Given that some fetal diagnoses may be imprecise and that the clinical condition can change after birth, postnatal evaluation is essential in guiding early management and treatment planning. Importantly, we had the opportunity to review the postnatal echocardiograms to better understand patient-level factors associated with both mortality and circulatory outcomes at the time of neonatal hospital discharge [9].

Antenatal echocardiography suggested a severe form of Ebstein’s anomaly with poor prognosis, which initially contributed to the decision for a DNAR order. However, postnatal echocardiography revealed a milder phenotype, characterized by preserved forward pulmonary artery flow and moderate to severe tricuspid regurgitation. This reassessment significantly altered the prognosis and supported continuation of active management.

The spectrum of Ebstein’s anomaly varies widely, ranging from mild forms, where neonates may remain stable with minimal intervention, to severe cases associated with cardiomegaly, poor right ventricular function, and high early mortality. These differences highlight the importance of postnatal reassessment to define the actual severity of the lesion and guide decision-making.

In neonatal cases of Ebstein’s anomaly, there is evidence that initial management with supportive therapies, such as prostaglandin E1 infusion, mechanical ventilation, or surgical interventions, can lead to stabilization. High long-term survival rates with acceptable rates of biventricular repair (BVR) can be achieved with proper RV assessment. Controlling pulmonary regurgitation in patients with anatomical pulmonary atresia (PA) is key [10]. This emphasizes the critical role of right ventricular function and pulmonary valve competence in determining prognosis. In Ebstein’s anomaly, the degree of pulmonary regurgitation can markedly affect oxygenation, cardiac output, and the risk of heart failure. Therefore, accurate postnatal evaluation of pulmonary flow dynamics is fundamental for guiding both immediate stabilization and long-term surgical planning.

These scenarios warrant reconsideration of pre-existing DNAR orders if the infant shows meaningful clinical improvement. Despite significant improvements in prenatal screening, fetal echocardiography is not always accurate for congenital heart defects. One study reported an overall accuracy of 87%, including six false negatives and four false positives out of 116 cases (sensitivity: 95%, specificity: ~87%) [11].

Given that some fetal diagnoses may be imprecise and that the clinical condition can change after birth, postnatal evaluation is essential in guiding early management and treatment planning. These insights reinforce the necessity for cautious, flexible clinical judgment in the neonatal period, as diagnostic accuracy improves and survival outcomes evolve, requiring protocols to adapt to the true postnatal condition of each neonate [12].

## Conclusions

Continual clinical reassessment in neonates with complex conditions is essential, especially when prenatal DNAR orders are in place. This case highlights the potential harm of rigid adherence to such early decisions without consideration of evolving clinical evidence. Early collaboration, ethical mindfulness, and a willingness to revise care plans are key to optimizing neonatal outcomes. The infant’s eventual discharge home on room air with both parents not only reflects favorable clinical progress but also underscores the profound impact of timely reassessment and multidisciplinary care in ethically complex neonatal scenarios.
